# New Fixed Assets Investment Project Environmental Performance and Influencing Factors—An Empirical Analysis in China’s Optics Valley

**DOI:** 10.3390/ijerph16244891

**Published:** 2019-12-04

**Authors:** Fumin Deng, Yanan Jin, Meng Ye, Shuangyi Zheng

**Affiliations:** Business School, Sichuan University, Chengdu 610065, China; dengfm@scu.edu.cn (F.D.); 2017325020041@stu.scu.edu.cn (Y.J.); 2017225020025@stu.scu.edu.cn (M.Y.)

**Keywords:** environmental performance evaluation, influencing factors, new fixed asset investment projects, energy conservation assessment and review

## Abstract

Civilization prospers when the ecology prospers, and civilization decays when the ecology decays. As an effective indicator of sustainable development of economy and resource environment, environmental performance can comprehensively reflect the actual level of coordinated development of the economy and environment system. This paper exemplifies China’s Optics Valley to evaluate its environmental performances and research the influencing factors of new fixed assets investment projects, for which a new fixed assets investment project environmental performance assessment system was constructed. The measurement model for the system was constructed using a generalized data envelopment analysis (DEA) with undesirable output to evaluate the environmental performances of the new fixed assets investment projects in China’s Optical Valley from 2011 to 2016. The multi-regression model with eight environmental performance hypotheses was established to determine the key influencing factors and to propose targeted countermeasures to enhance low-carbon innovation and promote low-carbon economic development. The results indicated that implementing new fixed assets investment project energy conservation assessments and reviews in high-tech areas could assist companies and the government in achieving better management.

## 1. Introduction

The industrial revolution brought scientific and technological progress to society; however, this rapid development also brought many problems, the most serious of which have been environmental pollution, the overexploitation of natural resources, and the destruction of ecological environments [1,2,3]. Environmental problems have become so serious today that they are threatening any attempts at sustainable socio-economic development. In response to this growing environmental crisis, “ecological priority” has become an important principle when formulating major development strategies. For example, on June 16, 2018, China promulgated the “Opinions of the CPC Central Committee and the State Council on Strengthening Ecological Environment Protection and Resolutely Fighting Pollution Prevention and Control”, in which the need to “implement the red line of ecological protection and firmly adhere to the ecologically sound development path of civilization” was particularly stressed. Natural environment pollution sources are primarily the result of careless human activities, which has been especially true of enterprise production activities [4]. Because enterprises have been and continue to be the main environmental polluters, they now must take responsibility for protecting the environment [5]. Further, as the public is becoming more aware of environmental governance issues, enterprise and government environmental performances and the associated influencing factors have become the focus of extensive research [6,7,8]. 

China’s main economic development models for the 21st century economy have been established in national high-tech development zones. Since September 2010, the energy conservation assessment and review system for China’s fixed assets investment projects has officially entered the implementation phase; therefore, research and analysis into the environmental performance of fixed assets investment in high-tech development zones and its influencing factors are going to inevitably play a pivotal role in the construction of China’s two-type society. China’s Optical Valley has proven to be an outstanding central and western region representative and has been ranked as one of the top 10 industrial parks in China. Therefore, by exemplifying China’s Optics Valley, this paper examines the internal and external factors associated with the environmental performance of new fixed assets investment projects as an example of low-carbon economic development, the results from which can provide a valuable reference for the formulation of corporate environmental management policies, the establishment of environmental management systems, and the development of environmental management practices, all of which can improve the operability of environmental policies and ensure the achievement of relevant ecological civilization deployment goals.

**Figure 1:**
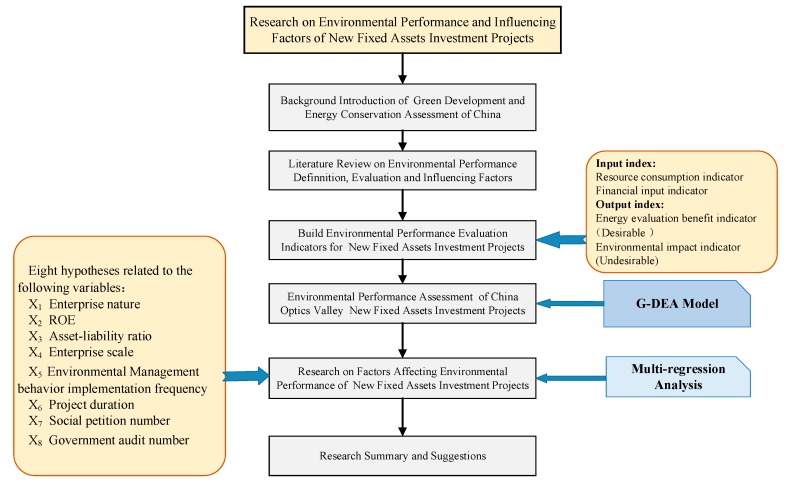
Article structure map.

The remainder of this article is organized as follows. Section 2 reviews and summarizes the literature on the definition of environmental performance, environmental performance evaluation methods, and influencing factors; Section 3 first establishes the environmental performance evaluation indicators for a fixed assets investment in new projects, and then uses a G-DEA model to evaluate the environmental performances of China’s Optical Valley’s fixed assets investment in new projects; Section 4 proposes eight hypotheses to study the factors affecting environmental performance and conducts associated regression analyses; and Section 5 summarizes the results, gives policy recommendations, and concludes the paper. The article structure is shown in Figure 1.

## 2. Literature Review

The primary focus of this paper is to evaluate the environmental performance of fixed assets investment in new projects and study the influencing factors. Therefore, this work is related to three main research streams: environmental performance definition, environmental performance evaluations, and the factors affecting environmental performance.

### 2.1. Environmental Performance Definition 

Environmental performance is basically a comprehensive systems concept that encompasses environmental financial and environmental management performance and the impact on the socio-economy. Therefore, as it is a relatively complex system concept, there have been varying definitions, with the most common being the economic value borne by an environmental load unit [9,10,11,12]. Ruf et al. and Carroll both defined environmental performance as the extent to which an enterprise meets stakeholder expectations of environmental responsibility [13,14]. Lankoski saw environmental performance as a vector that measured an enterprise’s impact on the environment [15,16], and Wood claimed that the principled expression of corporate social responsibility was a process related to the social input, production and output responses, with the social impact being reflected in environmental performance [16]. 

### 2.2. Environmental Performance Evaluation

Research on corporate environmental performance evaluation is mainly divided into the selection of evaluation indicators and the improvement of evaluation methods. Industrial pollution discharge evaluation indicators mainly include wastewater, exhaust gas, and solid waste [17]. Industrial wastewater includes production wastewater, sewage, and cooling water, which contains industrial production materials, intermediate products, by-products, and pollutants generated during the production process [18]. There are many types of pollutants released into the atmosphere from industrial production, including soot, sulfur oxides (such as SO_2_), nitrogen oxides (such as NO_2_), and carbon compounds (CO_2_) [19,20]. Industrial waste is a variety of waste residue, dust, and other wastes discharged into the environment during industrial production [21]. The above-mentioned indicators are enterprise environmental assessment indicators from a macro perspective, but there is currently no micro-level research on environmental performance assessment indicators for new fixed asset projects. A general research consensus has been reached that enterprise production sustainability should be measured with environmental performance evaluations. Most environmental performance research has been involved in updating algorithmic models and empirical analyses [22,23,24]. For example, the TRI (Toxics Release Inventory) has been a widely used aggregate index for land, water, and air emissions as it can compare company performances and reveal the effects of different regulations or economic tools [25,26]. Although there have been some informative studies in this area, shortcomings related to research object specialization and weak research method universality remain [27]. Another representative environmental performance evaluation method has been life cycle assessments (LCA); however, this had similar shortcomings [22]. Tyteca pointed out the deficiencies in existing environmental performance evaluation indicators and proposed a comprehensive environmental performance index that employed data envelopment analysis (DEA) [28]. To calculate relative efficiency, data envelopment analysis (DEA), which was first proposed by Charnes, has attracted significant research attention and continual expansion [29]. To avoid the subjectivity and randomness of current methodologies, most environmental performance researchers now prefer DEA when evaluating environmental performance [30,31,32]. 

### 2.3. Factors Affecting Environmental Performance

Most environmental performance research has been focused on determining the internal and external factors and identifying the related laws to verify the relationships between corporate environmental performance and financial performance [33,34,35,36]. For example, Yin and Ma found that the environmental standards adopted by enterprises could not be equated with their actual environmental performances [36], Dawkin and Fraas collected data from 500 S&P companies and proved that there was a U-type relationship between environmental performance and environmental information disclosure [37], Xiao et al. found that the social environment of a country affected enterprise environmental performances [38], Pineiro-Chousa, Romero-Castro, and Vizcaíno-González argued that socially responsible investment indices could provide a new analysis approach for assessing the relationship between corporate financial and corporate environmental performances [39], and Farooq found that employee participation had a strong and positive impact on corporate environmental performance [40]. Other studies have analyzed the relationships between environmental performance and industrial structure, international trade, and technology, and others have suggested specific environmental supervision, market structures, and lean production developments for management decision-making in China’s eco-industrial demonstration parks [41,42,43,44].

Even though there has been some valuable research conducted, these studies have had some limitations. First, as almost all have focused on overall industry environmental performance, the results are limited because the research covers a wide range of enterprises, periods, regions, environmental management characteristics, and environmental performances. Second, China is in an economic development and transformation stage from an extensive economy to an intensive economy, and therefore, economic growth relies more on the society itself than government policies or management. Third, there has been little recent research into environmental performances at high-tech development zones, and while the research on environmental performance influencing factors has moved from single factor to multiple factors measure. There have been few studies to date on the specific environmental performances of new projects and the associated internal and external influencing factors.

As environmental performance evaluations are the cornerstone of effective environmental management system operations, an analysis of the new project environmental performances in China’s Optical Valley could reveal the current state of corporate environmental management and the specific internal and external influencing factors, which could improve the applicability of environmental policies, and assist in the promotion of corporate environmental responsibility and the construction of an ecological civilization in high-tech zones.

## 3. New Fixed Assets Investment Project Environmental Performance Measurements

Environmental performance evaluations have their own particularities. First, the research enterprise environmental problems are essentially external, non-economic issues as high-tech zone enterprises conduct energy-saving assessments and review processes before project launches to improve energy efficiency and reduce external uneconomic influences. Second, environmental performance is reflected in the long-term interactions between corporate environmental management and resource utilization. While investment in environmental management and clean production technologies increases a project’s environmental costs in the short term, the comprehensive benefits to be derived from this investment in terms of environmental governance and the achievement of the environmental management objectives only becomes evident over the long term. Third, current corporate environmental management effects are diverse; that is, while the assessment of environmental performance is inseparable from environmental assessment indicators such as carbon dioxide emissions, it is also inseparable from financial input indicators, such as green fees and sewage charges.

### 3.1. Generalized DEA Model with Undesirable Output 

When using DEA to evaluate the production or operational efficiencies of an economic system, the larger the output, the better. However, discharged waste and pollutants do not follow these principles as the less waste and pollutants, the better it is for the enterprise production process. As the value of waste and pollutants cannot be zero because waste and pollutants from production processes cannot be avoided, when assessing the environmental performance of new high-tech zone projects, to evaluate the comprehensive efficiency, a generalized DEA model with undesirable outputs has the following advantages:

Firstly, as environmental performance is a complex system problem, the weights between the indicators are difficult to determine; however, a generalized DEA does not need the indicator weights to be determined in advance. Secondly, the complexity of environmental performance assessments makes it difficult to determine the quantitative relationships within the system; however, the generalized DEA method does not need the quantitative relationships within the system to be determined in advance. Thirdly, traditional DEA is unable to select the sample to evaluate the decision unit based on need, whereas the generalized DEA method is able to determine the reference plane as needed.

### 3.2. Model Construction and Validity Judgment

In this system model, it is assumed that there are *n* decision units and $\bar{n}$ sample units, with each unit being characterized by *m* input indicators, *s* kinds of desirable output indicators (expected growth indicators) and *k* kinds of undesirable output indicators (reflecting negative effects), with the values for the sample and decision units being positive numbers.

Let the input index value of the *j* sample unit be:

(1) $${\bar{x}}_{j}=({\bar{x}}_{{}_{1j}},{\bar{x}}_{{}_{2j}},\ldots ,{{\bar{x}}_{{}_{mj}})}^{T},$$

the desirable output indicator value be:

(2) $${\bar{y}}_{j}=({\bar{y}}_{{}_{1j}},{\bar{y}}_{{}_{2j}},\ldots ,{{\bar{y}}_{{}_{sj}})}^{T},$$

the undesirable output indicator value be:

(3) $${\bar{z}}_{j}=({\bar{z}}_{{}_{1j}},{\bar{z}}_{{}_{2j}},\ldots ,{{\bar{z}}_{{}_{kj}})}^{T}$$

Let the input index value of the *p* decision unit be:

(4) $$x_{p}=(x_{1p},x_{2p},\ldots ,{x_{mp})}^{T},$$

the desirable output indicator value be:

(5) $$y_{p}=(y_{1p},y_{2p},\ldots ,{y_{sp})}^{T},$$

the undesirable output indicator value be:

(6) $$z_{p}=(z_{1p},z_{2p},\ldots ,{z_{kp})}^{T}.$$

Let the sample unit set be:

(7) $$T^{*}=\left\{\left({\bar{x}}_{1},{\bar{y}}_{1},{\bar{z}}_{1}),\;({\bar{x}}_{2},{\bar{y}}_{2},{\bar{z}}_{2}),\ldots ,\;({\bar{x}}_{\bar{n}},{\bar{y}}_{\bar{n}},{\bar{z}}_{\bar{n}}\right)\right\},$$

the decision unit set be:

(8) $$T_{DMU}=\left\{\left(x_{1},y_{1},z_{1}),\;(x_{2},y_{2},z_{2}),\ldots ,\;(x_{n},y_{n},z_{n}\right)\right\}.$$

According to the idea of constructing production possible set by the DEA method, the possible production set determined by sample unit is as follows:

(9) $$\begin{array}{cc} T=\{(x,y,z)|\overset{\bar{n}}{\underset{j=1}{\sum }} & {\bar{x}}_{j}\lambda_{j}\\ & \leq x,\overset{\bar{n}}{\underset{j=1}{\sum }}{\bar{y}}_{j}\lambda_{j}\\ & \geq y,\overset{\bar{n}}{\underset{j=1}{\sum }}{\bar{z}}_{j}\lambda_{j}\leq z,\delta_{1}\left(\overset{\bar{n}}{\underset{j=1}{\sum }}\lambda_{j}+\delta_{2}{\left(-1\right)}^{\delta_{3}}\lambda_{\bar{n}+1}\right)=\delta_{1},\lambda_{j}\geq 0,j=1,2,\ldots ,\bar{n}+1\} \end{array}$$

$\delta_{1},\delta_{2},\delta_{3}$ are the parameters of 0 or 1.

And the set of production possibilities identified by the sample unit set be:

(10) $$\begin{array}{cc} T(d)=\{(x,y,z) & |\overset{\bar{n}}{\underset{j=1}{\sum }}{\bar{x}}_{j}\lambda_{j}\\ & \leq x,\overset{\bar{n}}{\underset{j=1}{\sum }}d{\bar{y}}_{j}\lambda_{j}\\ & \geq y,\overset{\bar{n}}{\underset{j=1}{\sum }}{\bar{z}}_{j}\lambda_{j}\leq z,\delta_{1}\left(\overset{\bar{n}}{\underset{j=1}{\sum }}\lambda_{j}+\delta_{2}{\left(-1\right)}^{\delta_{3}}\lambda_{\bar{n}+1}\right)=\delta_{1},\lambda_{j}\geq 0,j=1,2,\ldots ,\bar{n}+1\} \end{array}$$

*d* is a positive number and is called the shift factor.

Based on the G-DEA and G-DEAd efficiency concepts, a generalized DEA model (DGU) with undesirable output is constructed as follows:

(11) $$(DG_{U})\left\{ \begin{array}{l} \min\theta ,\\ s.t.\\ \theta x_{p}-\sum_{j=1}^{\bar{n}}{\bar{x}}_{j}\lambda_{j}\geq 0,\\ -y_{p}+\sum_{j=1}^{\bar{n}}d{\bar{y}}_{j}\lambda_{j}\geq 0,\\ z_{p}-\sum_{j=1}^{\bar{n}}{\bar{z}}_{j}\lambda_{j}\geq 0,\\ \delta_{1}(\sum_{j=1}^{\bar{n}}\lambda_{j}+\delta_{2}{\left(-1\right)}^{\delta_{3}}\lambda_{\bar{n}+1})=\delta_{1},\\ \lambda_{j}\geq 0,j\geq 1,2,\cdots \bar{n}+1 \end{array}\right..$$

When $\delta_{1}$ = 0, the model is based on the CCR model in a generalized DEA model with undesirable output. In calculating the environmental performance of New Fixed Asset Investment Projects involved in energy conservation assessment and review of high-tech zones, the CCR model in the generalized DEA model with undesirable output is needed.

### 3.3. Screening of Environmental Performance Evaluation Indicators for New Fixed Assets Investment Projects

After reviewing existing environmental performances, environmental protection statistics and related statistical yearbooks, academic papers, and research reports, this study determined the environmental performance evaluation indicators for the new projects from resources, economic, and environmental aspects, with the indicator selection being based on basic evaluation principles and energy conservation assessment and review reports to reflect resource consumption, pollution emissions, and energy savings after the assessment [44].

The assessment indicators are divided into three levels. The input indicators are the resource consumption indicators and the financial input indicators, with the resource consumption index being composed of power consumption, water consumption, steam consumption, and natural gas consumption, and the financial input indicator being total investment. The output indicators are divided into desirable output indicators and non-desirable output indicators, with the desirable output indicators being the performance evaluation indicators that correspond to the energy-saving indicator in the energy-saving assessment reports, and the undesirable output indicators being the industrial waste gas emissions resulting from the project implementation, such as carbon dioxide and sulfur dioxide, which also reflect the environmental impact of new regional construction projects. The indicator settings are shown in Table 1.

### 3.4. Environmental Performance Assessment Process and Results Analysis 

The DEA algorithm requires that the number of Decision Making Units (DMUs) be no less than the number of input indicators multiplied by the output indicators or no less than three times the sum of the input and output indicators [45]:

N ≥ max{X*Y, 3*(X + Y)}(12)

In this study, X = 5 is the number of input indicators, and Y = 3 is the number of output indicators. The input indicators are projected power consumption (10,000 kwh), projected natural gas consumption (10,000 m^3^), projected water consumption (10,000 m^3^), projected steam consumption (10,000 t) and projected total investment (10,000 Chinese Yuan (CNY)), and the output indicators are the expected energy savings (tonnes of standard coal), and expected carbon dioxide emissions (tonnes) and sulfur dioxide emissions (tonnes). To ensure the accuracy of the final result, equivalent values are taken for the three output indicators.

Because the basic requirement for running the DEA algorithm is that N conforms with equation (12), 24 is selected as the standard reference value (3 × 5 = 15 and 3 × (5 + 3) = 24). As the overall sample number 237 was much larger than the standard reference value, the above algorithm could be executed.

**Figure 2:**
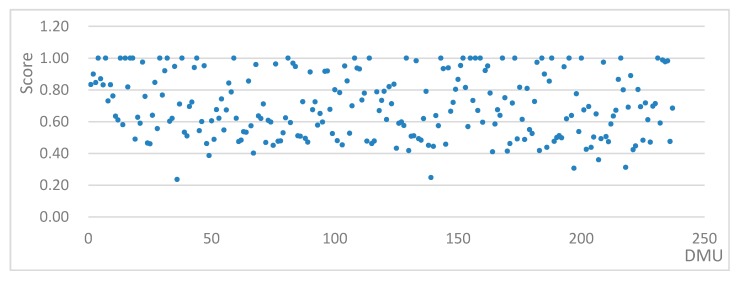
Environmental performance value distribution map.

To calculate the environmental performance for new high-tech zone fixed asset investment projects, the input-oriented CCR model was combined with an SBM super-efficiency model and then run on the MAXDEA PRO 6.0 tool to determine the static environmental performance values for each new project. The specific distribution is shown in Table 2, and a more intuitive view of the environmental performance value distribution is plotted and shown in Figure 2.

## 4. Factors Affecting the Environmental Performance of New Fixed Assets Investment Projects 

### 4.1. Research Hypothesis

Based on the literature review in Section 2, it was surmised that environmental performance is affected by both internal and external factors. The internal environmental performance factors are the enterprise size, the environmental awareness of the enterprise manager, the pollution attributes of the industry in which the enterprise is located, the current enterprise technical level, the financial health status, the nature of the enterprise, the product attributes and services provided, the environmental technology investment proportion, and the ISO environmental certification. As different internal factors affect environmental performance, the project feedback on environmental pressures and associated environmental project costs are different, which means that each project has different environmental behaviors and performances. The external factors affecting environmental performance are government rules and regulations, consumer demand for environmentally friendly products, public supervision of corporate management environmental behavior, attention by the media and other non-government organizations to corporate environmental information disclosure, imitation competition behavior among competitors in the same industry, investment selection criteria, and the upstream and downstream enterprise trade agreements, all of which have different driving force or external pressure effects on enterprise environmental performance. Therefore, based on the above and combined with the field research results, this paper proposes the following eight hypotheses.

**Hypothesis** **1.** *There is a positive correlation between the nature of the project owner’s business and the environmental performance of new projects.*

If the new project enterprise is a listed company, it has more social responsibility than a non-listed enterprise as the shareholders of listed companies can influence internal environmental management decisions [46]. Further, as listed companies also need to publish a “Corporate Social Responsibility Report” or an independent environmental report every year [40], enterprises with high information disclosure levels need to pay more attention to environmental protection and environmental performance improvements.

**Hypothesis** **2.** *There is a positive correlation between project profitability and new project environmental performance.*

Enterprises seek profitability, which is a reflection of their capital appreciation; that is, in a normal business year, profitability allows an enterprise to further expand and re-invest its accumulated assets [33,34,35,36]. Companies with strong profitability often have more resources, higher quality products, stable markets, harmonious partnerships, and a growing development space; therefore, they are often more willing to put more resources into energy conservation, emissions reduction, and environmental management protection, with the objective of improving their environmental performance and establishing a good ecological image.

**Hypothesis** **3.** *There is a negative correlation between enterprise financial status and new project environmental performance.*

Based on previous research [36,47], the higher the enterprise debt, the lower the environmental management enthusiasm, and the lower the asset-liability ratio, the higher the enterprise’s environmental management enthusiasm. As China is still a developing country, there will be a continuing focus on economic development for some time. Therefore, enterprise production and operations are mainly aimed at reducing controllable costs, increasing marginal revenue, achieving a maximum net profit, or pursuing maximum benefit, all of which means that most companies tend to pay less attention to environmental performance than to meeting local government economic goals. Consequently, only profitable enterprises take responsibility for environmental management as enterprises with high asset-liability ratios find it difficult to implement consistent environmental management strategies.

**Hypothesis** **4.** *There is a positive correlation between enterprise scale and new project environmental performance.*

The theory of scale economy and management economic market structure states that the larger the enterprise scale, the greater its influence in the market. To avoid monopoly, however, large-scale enterprises are subject to greater government regulation and are more affected by government environmental policies, regulations, and standards [39,40]. Second, the larger the enterprise, the more attention the daily business activities receive from stakeholders. Therefore, as business owners or managers must consider the behavior of the government, the media, and the public when formulating corporate strategy, to attract investors and consumers and ensure a good industry reputation, most take the initiative to undertake their social environmental responsibilities and obligations.

**Hypothesis** **5.** *There is a positive correlation between environmental management behavior implementation frequency and the environmental performance of new projects.*

Enterprise environmental management in this paper is seen to be part of strategic enterprise management. By implementing environmental management as part of everyday corporate behavior, the corporate costs and public welfare losses caused by environmental problems can be minimized. Therefore, the extent to which corporate environmental management is integrated in enterprise strategies is an indicator of enterprise environmental management [37,47]. For example, joining environmental protection organizations, establishing special environmental management departments, and implementing new energy conservation and emissions reduction technologies to reduce environmental pollution and resource waste can all improve the environmental performance of new projects.

**Hypothesis** **6.** *There is a positive correlation between the project duration and the environmental performance of new projects.*

It has been observed that the longer the project construction period, the greater the total project input, and that most projects with long construction periods have complex technical standards and/or are difficult to construct. Therefore, the energy-saving technologies and energy-saving standards used in project construction are described in detail in the respective energy-saving assessment and review reports. Further, projects with long project construction periods have more government and public supervision than projects with shorter construction periods. Therefore, the longer the construction, the greater the external pressure, and the higher the environmental performance.

**Hypothesis** **7.** *There is a positive correlation between the frequency of society petitions and the environmental performance of new projects.*

**Hypothesis** **8.** *There is a positive correlation between government supervision and the environmental performance of new projects.*

Hypotheses 7 and 8 are related to many research results that indicated that corporate environmental performance management was influenced by the environmental behavior of the government environmental departments, investors, and the public [38,43,46,48]. In recent years, the government, society, and the public have demanded better environmental protection and have insisted that for any new fixed asset investment projects, enterprises must ensure environmental protection to stop the common enterprise free-riding behaviors. In these circumstances, the government is a vital independent third-party supervisor to ensure proper corporate environmental management and insist on the control of external factors by high-tech zone management committees to encourage enterprises to pay attention to and continuously improve their environmental management, reduce their environmental pollution, and improve their resource utilization. Therefore, this study believes that the supervision of public and government agencies improves the environmental performance of new projects.

### 4.2. Variable Design

In this paper, the environmental efficiency (score), which is between (0, 1) (including 1, and excluding 0), calculated using the DEA is the dependent variable Y for the new project environmental performance; the closer the score is to 1, the higher the resource allocation efficiency of the new project, and the higher the environmental management level of the project, the better the environmental performance. Therefore, 1 indicates efficiency; from the relative efficiency analysis, that is, 100% of the resource inputs contribute to the output, and there is almost no resource waste.

The independent variables are the internal and external factors that affect environmental performance. In actual situations, there may be certain correlations between the internal and external factors that affect the environmental performance of new projects. Therefore, the independent variable index is set with the internal influencing factors mainly based on the financial indicators, and the external influencing factors mainly based on the external stakeholders. 

Therefore, the internal influence factors used in this paper are as follows:

(1) The nature of the enterprise (Is it a listed company? Yes = 1; no = 0);
(2) Corporate profitability (return on net assets, indicating the ability of the net assets to generate profits);
(3) Financial status (the asset-liability ratio is an effective indicator for evaluating the financial health of enterprises, and is also an indicator that can measure the ability of enterprises to use creditor funds for reasonable business activities);
(4) Enterprise scale (registered capital of the enterprise, <10 million CNY = 1, 10–50 million CNY = 2, 50–100 million CNY = 3, 100–500 million CNY = 4, 500 million–1 billion CNY = 5, >1 billion CNY = 6);
(5) Implementation frequency of environmental management behavior (based on the relevant national environmental policies, regulations, and standards (Table 3);
(6) Construction period (the time span from the start of construction to the completion of the project, by month).

The external influence factor independent variables are:

(7) The number of letters and visits from social groups;
(8) The number of supervisory actions taken by the government.

### 4.3. Data Sources

To determine the environmental performance influencing factors for new high-tech zone projects, enterprise environmental management professionals, and related front-line staff, including middle and high-level management personnel, enterprise production departments, project construction enterprise working groups, and enterprise pollution treatment departments, were consulted. 

The following additional data was collected. Basic data from 2011 to 2016 and some data related to the internal operations of some enterprises were collected from the low-carbon development and enterprise clean production soft science research base and clean production center. If the enterprise was listed, its annual report, corporate website information, and corporate social responsibility reports were consulted. Relevant data on business durations and operations, total assets, net assets, net profit, environmental protection expenditures, and scientific research investment were extracted from the Wuhan Municipal Bureau of Statistics and the “Compilation of Key Enterprises in Donghu High-tech Zone” compiled by the Statistical Center of Wuhan East Lake High-tech Development Zone. Data on new project environmental management implementation frequencies, the pollution attributes for the industries to which the enterprises belonged, the number of government supervisory visits and audits, and the number of mass petitions were extracted from energy conservation assessment reports and questionnaires. After the statistical analysis, a dummy variable was set. The variable definitions, indicator selections, and data type selections are detailed in Table 4.

### 4.4. Model Selection and Result Analysis

The environmental performance for new high-tech zone projects and the associated influencing factors were developed as a general form of the regression model based on the preliminary research hypothesis. The general form was expressed as:

(13) $$Y=\beta_{0}+\beta_{1}X_{1}+\beta_{2}X_{2}+\beta_{3}X_{3}+\beta_{4}X_{4}+\beta_{5}X_{5}+\beta_{6}X_{6}+\beta_{7}X_{7}+\beta_{8}X_{8}$$

where Y is the environmental performance value of the dependent variable calculated by DEA, $\beta_{i}\left(i=1,2,3,4,5,6,7,8\right)$ is the regression coefficient for the model, *X*_1_–*X*_8_ are the independent variables (eight internal and external influencing factors), and $\beta_{0}$ is a constant term.

#### 4.4.1. Descriptive Statistical Analysis

The descriptive statistics in Table 5 indicate that the mean value for the Y environmental performance was 0.6976; that is, 69.76% of environmental resource inputs contributed to the environmental output, but 30.24% of the environmental resources investment was wasted in the implementation process, which means that the environmental performance level of the new project had a large improvement space. The standard deviation for the environmental performance was 0.1985, which was less than 0.2, which indicated that the environmental performance was concentrated around the mean. The minimum environmental performance value was 23.7%, and the maximum was 100%, which meant that the overall environmental performance evaluation results were different for different projects, that there were significant environmental performance differences in the new projects, and that the environmental performances across the enterprises were uneven. 

The mean enterprise nature data group indicator indicated that there were more non-listed enterprises than listed enterprises in the research sample. The ROE average was 0.1598, the maximum was 2.68, and the minimum was −1.25. The mean value was low, and the maximum value and the minimum value had positive and negative values, indicating that the new project owner had a win or loss in their current business. The asset-liability ratio, which reflected the long-term solvency, was generally low, but the financial status was relatively healthy, with an average of 0.487 and a standard deviation of 0.256, which indicated that the asset-liability ratio was concentrated around the mean. The enterprise scale also adopted dummy variables, which were counted based on the registered capital scale of the project; that is, a minimum value of 1, a maximum value of 6, and an average value of 3.18; therefore, the enterprise size distribution was considered reasonable. The four variables for environmental management: implementation frequency, project duration, social petitions, and government audits, were extracted from actual statistics. In the sample, the enterprises that had environmental management system certifications, environmental protection departments, and new energy conservation and emissions reduction technologies were counted as the environmental management behavior implementation frequencies; the maximum environmental management behavior implementation frequency was 37, the minimum value was 3, and the average was 19.36; therefore, environmental management for the new project fixed assets investment was considered good. The project duration was based on a monthly construction period, with the maximum being 83, the minimum being 2, the mean being 28.05, and the standard deviation was 13.24, which indicated that the sample data in this group were relatively dispersed, which was consistent with the actual situation because new project scales, technical implementation difficulties, and therefore construction periods varied considerably. The number of social petitions was taken as the number of complaints from residents in surrounding communities during new project construction, for which the maximum was eight, the minimum was zero, and the mean was 2.56. The number of government audits was the number of times an enterprise was audited by the environmental department, for which the maximum was 5, the minimum was 1, and the mean was 3.31. All indicators in the above data sets reflected the actual new project situation for enterprises currently participating in energy conservation assessments and reviews.

#### 4.4.2. Statistical Tests and Regression Analyses

In this paper, SPSS Statistics for Windows, version 19.0 ( SPSS Inc. Chicago, IL, USA) was used to calculate the Pearson’s correlation coefficient to test the correlations between the explanatory variables.

The correlation matrix in Table 6 shows the strength and direction of each independent variable and dependent variable, as well as the correlations between the independent variables. As can be seen, all eight independent variables were correlated with Y, most indicators passed the 1% (bilateral) and 5% (bilateral) significance tests, and the eight selected independent variables were found to better explain the dependent variables. The correlation between X_4_ (Enterprise scale) and X_6_ (Project duration) was 0.459 at a 0.01 significance level; therefore, as the absolute correlation value was greater than 0.4, there was a moderate or low degree of correlation, indicating possible multicollinearity between the two indicators. Therefore, the multicollinearity in the multiple regression analysis was tested with Tolerance and VIF (variance inflation factor). As outlined in the fourth edition of modern psychology and educational statistics, the multiple regression method, which can be divided into a forced entry method and forced elimination method, should include all predictive variables in the regression equation when estimating the dependent variables. As the forced entry method includes all predictors with explanatory power for the dependent variables in the regression equation and then calculates the regression coefficients of all the variables without considering the relationships between the predictors at a certain significance level, the forced entry method was adopted in this paper, and therefore, there was only one regression model. As can be seen from the results for the relevant indicators in the fitting model in Table 7, the R square was 0.401, the Adjust R square was 0.349, and the goodness of fit was 0.349, which is considered a good result in social science and management economics. The results indicated that the eight independent variables in the regression model explained 34.9% of the degree of variation in the dependent variables.

Table 8 shows the variance analysis test results for the fitted regression model. The F-value for the significance test of variance was 187.677, and the P-value for the significance test was 0.000, which was less than the significance level of 0.05, indicating that the overall explanatory variance of the regression model reached the significance level. The F-value test also passed the significance test. The regression equation was found to have a good goodness of fit, and therefore, the regression equation reflected the research phenomenon well. However, as the statistical significance of the model did not mean that all model variables had statistical significance, it was necessary to further test each independent variable to determine which regression coefficients were significant. 

Therefore, a collinearity diagnosis was conducted on the multiple linear regression analysis, the results for which are shown in Table 9, which shows the regression coefficients in a coefficient summary table, the t-values, the significance probability values for the corresponding significance test, the tolerance, and the VIF for the collinearity statistics. The smaller the tolerance, the higher the accuracy of the prediction by the other independent variables, and the more serious the multicollinearity, and when the tolerance is less than 0.1, severe multicollinearity exists. The VIF (variance inflation factor) is the reciprocal of tolerance; that is, the larger the VIF, the more serious the multicollinearity; therefore, the VIF should not be greater than five in general. 

As can be seen from Table 9, the tolerance values for the eight independent variables were all greater than 0.1, and the VIF values were all below 4.000, with none being greater than the evaluation index value 10, which indicated that there were no obvious multicollinearity problems between the independent variables in the regression equation.

#### 4.4.3. Discussion

From Table 9, standardized regression coefficients were adopted for the standardized regression model, as follows:

(14) $$\begin{array}{c} Y=0.122*X_{1}+0.133*X_{2}-0.012*X_{3}-0.034*X_{4}+0.381*X_{5}-\\ 0.059*X_{6}-0.279*X_{7}+0.34{*X}_{8}, \end{array}$$

from which the following was observed.

(1) As the enterprise nature coefficient was 0.122, the symbol was positive, the t-test was 2.899, and the probability P was 0.027, it passed the 5% significance level test, which indicated that there was a significant positive correlation between environmental performance and enterprise nature; therefore, Hypothesis 1 was valid; that is, there is a positive correlation between the enterprise nature and the new project environmental performance.

(2) The ROE coefficient was 0.133, the symbol was positive, the t-test was 3.625, and the probability P was 0.032; therefore, the ROE passed the 5% significance test, which indicated that the environmental performance was positively correlated with ROE and Hypothesis 2 was valid; that is, there is a positive correlation between profitability and new project environmental performance.

(3) The asset-liability ratio coefficient was 0.012, the symbol was negative, the t-test was −2.015, and the probability P was 0.007, which passed the 1% significance test, indicating that environmental performance had a significant negative correlation with the asset-liability ratio and Hypothesis 3 was valid; that is, there is a negative correlation between the financial status of the project and the environmental performance of the new project.

(4) The enterprise scale coefficient was 0.034, the symbol was negative, the t-test was −1.148, and the probability P was 0.252; therefore, the significance test was not passed, which may have been due to the principle of managing diminishing returns and the limited enterprise scales. As the enterprise scale expands, transaction costs increase. At the same time, the management capacity was limited, and management efficiency too large, resulting in an efficiency decrease; therefore, Hypothesis 4 was not valid; that is, there is not a positive correlation between the project enterprise scale and new project environmental performance.

(5) The environmental management behavior implementation frequency coefficient was 0.381, the symbol was positive, the t-test was 8.397, and the probability *P* was 0.000, which passed the 1% significance test, which indicated that there was a significant positive correlation between the environmental performance and the environmental management behavior implementation frequency; therefore, Hypothesis 5 was valid; that is, there is a positive correlation between the environmental management behavior implementation frequency and the environmental performance of new projects.

(6) The project duration coefficient was 0.059, the symbol was negative, the t-test was 3.506, and the probability P was 0.712; therefore, the significance test was not passed, which may have been related to social inertia. Because of the economic externalities associated with environmental problems, perfunctory behavior or free-riding is common. Therefore, the longer the project duration, the more obvious the perfunctory free-riding behavior, and the lower the environmental performance; therefore, Hypothesis 6 was not valid: that is, there is not a positive correlation between the project period and the environmental performance of the new project.

(7) The social petition coefficient was 0.279, the symbol was negative, the t-test was −7.449, and the probability *P* was 0.000, which passed the 1% significance test, and indicated that the environmental performance level had a significant negative correlation with the number of social petitions; therefore, Hypothesis 7 should be corrected to the following; there is a negative correlation between the society petition frequency and the environmental performance of new projects. The reason for this could be that the amount of pollution discharged by new projects participating in energy conservation evaluation assessment and reviews does not normally reach the evaluation standard, which can significantly impact the surrounding communities; therefore, there would be an increase in opposition and complaints. 

(8) The government audit number coefficient was 0.340, the symbol was positive, the t-test was 8.010, and the probability *P* was 0.000, which passed the 1% significance test, and indicated that the environmental performance had a significant positive correlation with the number of government audits; therefore, Hypothesis 8 was valid: that is, there is a positive correlation between government supervision behavior and the environmental performance of new projects. The verification of this hypothesis indicated that, to a certain extent, external pressure (mainly from the management committee of the high-tech zone and local governments at various levels) could prompt enterprises to strengthen their environmental management behavior and improve their environmental performance. Therefore, high-tech development zone administrative committees need to supervise the environmental management of enterprises and carry out energy conservation evaluation assessments and reviews.

## 5. Conclutions

The evaluation index for new fixed asset investment projects, which included resources, economic, and environmental characteristics, was determined based on current research, energy conservation evaluations, and work reports. This paper exemplified China’s Optics Valley to develop an environmental performance evaluation method for new fixed assets investment projects and to identify the influencing factors, for which a system measurement model was constructed using generalized DEA with undesirable output, and a multiple regression analysis model established to determine the key internal and external influencing factors for environmental performance of new projects, from which the following conclusions were made.

(1) It was found that the average environmental performance for new high-tech zone projects was about 69.76%, which indicated that there was a certain degree of resource waste and a large environmental performance improvement space. A big difference was also found between the environmental performances for the various new projects. Therefore, it is recommended that enterprises implement clean production through process improvements and technological innovations, improve energy utilization efficiencies, and reduce unnecessary energy consumption and emissions. To improve the environmental performance of new projects and achieve sustainable development, enterprises should have the courage to assume corporate social responsibility and participate in energy conservation assessments and reviews. The government and relevant departments should also strengthen their environmental supervision and law enforcement, pay greater attention to the differences among different projects, and encourage enterprises to increase their environmental protection investment. 

(2) The influencing factor analysis results found that there were different countermeasures and suggestions based on the internal and external factors. 

As the enterprise nature, ROE and environmental management behavior implementation frequency internal factors were all found to have a positive impact on the environmental performance of new projects, and enterprise managers could consider the following aspects for improvements. First, non-listed enterprises should learn from listed enterprises and publish CSR reports or independent environmental reports every year to improve their environmental protection information transparency and CSR information disclosure quality. Second, as enterprises with higher profitability are better able to implement environmental governance and protection by increasing environmental protection investment, enhancing scientific and technological innovation, and implementing energy conservation and emissions reduction, they could stimulate environmental protection motivation and improve overall environmental performance. Third, the more frequently environmental management strategies are implemented, the higher the environmental performance of new projects; therefore, managers should pay attention to the development of a low-carbon development atmosphere by focusing on employee green development demands and responding to green and sustainable development requirements. Finally, the asset-liability ratio was found to have a negative effect on the environmental performance of new projects, which may indicate that high asset-liability ratio enterprises could face higher operating costs or energy conservation and emissions reduction technology implementation bottlenecks. Therefore, these enterprises should actively seek technical assistance and strengthen their communication with industry peers. The Chinese government and relevant departments could also provide greater tax incentives, set up special assistance funds, or provide technical assistance to high asset-liability ratio enterprises.

The external factors cover social management and government supervision. It was found that the larger the numbers of social petitions, the worse the environmental performance, which indicated that the number of social petitions could possibly reflect the actual environmental performance level of new projects, which in turn demonstrates that as public environmental awareness has grown, this could be used to encourage high-tech enterprises to implement environmental management strategies and improve new project environmental performances. To some extent, the level of public environmental awareness reflects the level of a country’s cultural civilization; that is, the higher the public’s recognition of environmental protection, the more quickly and efficiently it helps the government enact and implement environmental laws and regulations and the more enterprises are motivated to take active measures to improve their environmental management. Further, as strengthening government supervision and auditing was found to be associated with improved new project environmental performances, it is necessary to strengthen the environmental management audit of enterprises in high-tech zones by including energy conservation performance assessments and environmental protection performance assessments in the high-tech development zone management committee performance assessments. Further, to enhance the sense of responsibility for the relevant subjects and ensure the full implementation of energy conservation evaluation and review systems, the government performance appraisal department should seek to promote the implementation of a target responsibility system, establish an environmental protection special GDP assessment system, and include energy consumption indicators in the comprehensive evaluation system.

## Figures and Tables

**Table 1:** Environmental performance new project assessment indicators.

Target Layer	Input/Output Indicator			Specific Indicators
Environmental impact indicators	Input index		Resource consumption indicators	Power consumption
				Water Consumption
				Steam consumption
				Natural gas consumption
			Financial input indicator	Total investment
	Output index	Desirable output	Energy evaluation benefit indicator	Energy saving quantity
		Undesirable output	Environmental impact indicators	Carbon dioxide emissions
				Sulfur dioxide emissions

**Table 2:** Environmental performance values for the new fixed assets investment projects.

DMU	Score	DMU	Score	DMU	Score	DMU	Score	DMU	Score	DMU	Score
1	0.83403	41	0.69529	81	1	121	0.61373	161	0.92160	201	0.67344
2	0.89979	42	0.72310	82	0.59394	122	0.82040	162	0.95035	202	0.42593
3	0.84619	43	0.94080	83	0.96806	123	0.71306	163	0.78017	203	0.69537
4	1	44	1	84	0.94723	124	0.83562	164	0.41045	204	0.43802
5	0.87032	45	0.54297	85	0.51122	125	0.43246	165	0.58532	205	0.50311
6	0.83144	46	0.60018	86	0.50787	126	0.58977	166	0.67552	206	0.64870
7	1	47	0.95178	87	0.72552	127	0.59848	167	0.63967	207	0.35988
8	0.73059	48	0.46232	88	0.49472	128	0.57591	168	1	208	0.49269
9	0.83246	49	0.38664	89	0.47128	129	1	169	0.75094	209	0.97435
10	0.76183	50	0.60499	90	0.91254	130	0.41780	170	0.41471	210	0.50609
11	0.63478	51	0.48843	91	0.67503	131	0.50822	171	0.46198	211	0.47409
12	0.61079	52	0.67561	92	0.72500	132	0.51113	172	0.71713	212	0.58472
13	1	53	0.62132	93	0.57777	133	0.98343	173	1	213	0.63503
14	0.58068	54	0.74319	94	0.65162	134	0.49273	174	0.49167	214	0.67161
15	1	55	0.54714	95	0.59773	135	0.48347	175	0.81647	215	0.86567
16	0.81828	56	0.67385	96	0.91689	136	0.61918	176	0.61432	216	1
17	1.00000	57	0.84309	97	0.91936	137	0.79036	177	0.48824	217	0.80032
18	1	58	0.78713	98	0.67761	138	0.45114	178	0.80943	218	0.31238
19	0.48971	59	1	99	0.52481	139	0.24850	179	0.55034	219	0.69154
20	0.62746	60	0.62164	100	0.80127	140	0.44438	180	0.52532	220	0.88936
21	0.59018	61	0.47440	101	0.48057	141	0.63795	181	0.72654	221	0.42359
22	0.97459	62	0.48341	102	0.78251	142	0.57442	182	0.97385	222	0.44712
23	0.75880	63	0.53685	103	0.45369	143	1	183	0.41826	223	0.80231
24	0.46537	64	0.53311	104	0.95014	144	0.93345	184	1	224	0.69384
25	0.46057	65	0.85563	105	0.85613	145	0.45773	185	0.89982	225	0.48331
26	0.64031	66	0.57385	106	0.52725	146	0.93844	186	0.43862	226	0.71790
27	0.84698	67	0.40228	107	0.69964	147	0.66554	187	0.85442	227	0.61203
28	0.55617	68	0.95948	108	1	148	0.72128	188	1	228	0.47119
29	1	69	0.63619	109	0.93713	149	0.80350	189	0.47609	229	0.69730
30	0.76708	70	0.61983	110	0.93170	150	0.86606	190	0.49977	230	0.71354
31	0.92010	71	0.71120	111	0.73578	151	0.95307	191	0.51154	231	1
32	1	72	0.46924	112	0.77910	152	1	192	0.49861	232	0.59157
33	0.60245	73	0.60836	113	0.47749	153	0.81464	193	0.94534	233	0.98787
34	0.62087	74	0.59793	114	1	154	0.56920	194	0.61785	234	0.97681
35	0.94771	75	0.45065	115	0.46239	155	1	195	1	235	0.98257
36	0.23667	76	0.96320	116	0.47798	156	0.73294	196	0.63867	236	0.47521
37	0.71091	77	0.47641	117	0.78758	157	1	197	0.30682	237	0.68523
38	1	78	0.47882	118	0.66981	158	0.67038	198	0.77594		
39	0.53313	79	0.53002	119	0.73356	159	1	199	0.53735		
40	0.51001	80	0.62453	120	0.79115	160	0.59592	200	1		

**Table 3:** Some examples of enterprise environmental management.

No.	Environmental Management Examples	Relevant Legal Basis
1	The enterprise regularly conducts environmental training for employees	Environmental protection law of the People’s Republic of China; Environmental impact assessment law of the People’s Republic of China; Law of the People’s Republic of China on energy conservation; Regulations on the administration of environmental protection for construction projects.
2	Pollutant emissions enterprise system monitoring indicators	
3	The enterprise regularly publishes environmental reports	
4	The enterprise has implemented an environmental plan	
5	The enterprise has clear environmental requirements for the raw materials and suppliers.	
6	Extensive publicity and education on environmental protection and energy saving	
7	The enterprise’s products have an ISO9001 quality management system certification.	
8	The enterprise’s products have an ISO14001 environmental management system certification.	
9	The enterprise has a dedicated department or dedicated staff responsible for environmental management	
10	The enterprise has established an environmental management system with the supplier	
11	The enterprise purchases environmentally friendly equipment	
12	The enterprise evaluates environmental performance based on policy objectives and seeks reasonable improvements	
13	The enterprise has a clean production audit	
14	The enterprise has a documented environmental policy to guide environmental management	
15	The enterprise’s products have obtained GB/T18001 occupational health and safety management system certification	

Data source: the enterprise environmental management examples were mainly derived from the relevant national environmental policies, regulations, and standards.

**Table 4:** Variable definitions, indicator selections, and data types.

Variable Type	Variable	Variable Indicator Selection	Abbeviation
Dependent variable	Environmental performance	(0, 1]	Y
Independent variable	Enterprise nature	Listing = 1, no listing = 0	X_1_
	ROE	Roe = net profit/net assets	X_2_
	Asset-liability ratio	Asset-liability ratio = total liabilities/total assets	X_3_
	Enterprise scale	Registered capital	X_4_
	Environmental Management behavior implementation frequency	IFEMB, the data comes from questionnaire statistics	X_5_
	Project duration	The length of months from the start of construction to completion	X_6_
	Social petition number	Number of complaints and letters from residents of surrounding communities	X_7_
	Government audit number	Number of times the enterprise was audited by the environment during the project construction period	X_8_

**Table 5:** Descriptive statistics.

Variable	N	Minimum Value	Maximum	Mean	Standard Deviation
Y Environmental performance	237	0.23667	1.000	0.69759	0.198500
X_1_ Enterprise nature	237	0	1	0.03	0.170
X_2_ ROE	237	−1.25	2.68	0.1598	0.36811
X_3_ Asset-liability ratio	237	0.011	1.211	0.48746	0.255657
X_4_ Enterprise scale	237	1	6	3.18	1.336
X_5_ Environmental management behavior implementation frequency	237	3	37	19.36	7.556
X_6_ Project duration	237	2	83	28.05	13.240
X_7_ Social petition number	237	0	8	2.56	1.842
X_8_ Government audit number	237	1	5	3.31	1.117
Valid N (list state)	237				

**Table 6:** Pearson’s correlation analysis for the explanatory variables.

	Y	X_1_	X_2_	X_3_	X_4_	X_5_	X_6_	X_7_	X_8_
Y	1								
X_1_	0.127 *	1							
X_2_	0.119 *	−0.041	1						
X_3_	−0.129 *	−0.050	0.052	1					
X_4_	−0.019 *	−0.062	0.015	0.047	1				
X_5_	0.175 **	0.127	−0.107	−0.033	−0.141 **	1			
X_6_	−0.088 **	−0.103	−0.023	−0.031	0.459 **	−0.182 **	1		
X_7_	−0.216 **	−0.067	0.102	0.036	0.112 **	−0.047 **	0.169 **	1	
X_8_	0.155 **	0.019	−0.155	−0.049	−0.054 *	0.295 **	−0.148	−0.315 **	1

** Significantly correlated at the 0.01 level (both sides). * Significantly correlated at the 0.05 level (both sides). a. List *N* = 237.

**Table 7:** Model summary.

Model	R	R Square	Adjust R Square	Standard Estimated Error
1	0.633 ^a^	0.401	0.349	5.85059

^a^ Predicted variables: (constant), X_8_ government audit number, X_1_ enterprise nature, X_3_ asset-liability ratio, X_2_ ROE, X_6_ project duration, X_4_ enterprise scale, X_7_ social petition number, X_5_ environmental management behavior implementation.

**Table 8:** Regression equation F value.

Model		Sum of Square	Df	Mean Square	F	Sig.
1	Regression	8.073	8	1.009	187.677	0.000 ^a^
	Residual	1.226	228	0.005		
	Total	9.299	236			

^a^ Predicted variables: (constant), X_8_ government audit number, X_1_ enterprise nature, X_3_ asset-liability ratio, X_2_ ROE, X_6_ project duration, X_4_ enterprise scale, X_7_ social petition number, X_5_ environmental management behavior implementation.

**Table 9:** Results of the regression equation.

MODEL	Non-Standardized Coefficient		Standard Coefficient	t	Sig.	Collinear Statistic	
	B	Standard error	trial version			Tolerance	VIF
(constant)	0.392	0.037		10.512	0.000		
X_1_ Enterprise nature	0.126	0.029	0.122	2.899	0.027	0.953	1.050
X_2_ ROE	0.101	0.021	0.133	3.625	0.032	0.969	1.032
X_3_ Asset-liability ratio	−0.010	0.019	−0.012	−2.015	0.007	0.988	1.012
X_4_ Enterprise scale	−0.005	0.004	−0.034	−1.148	0.252	0.654	1.529
X_5_ Environmental management behavior implementation frequency	0.010	0.001	0.381	8.397	0.000	0.281	3.560
X_6_ Project duration	−0.33	0.055	−0.059	3.506	0.712	0.777	1.287
X_7_ Social petition number	−0.030	0.004	−0.279	−7.449	0.000	0.411	2.433
X_8_ Government audit number	0.060	0.008	0.340	8.010	0.000	0.321	3.118
